# Inhibitory Effects of Compounds Isolated from *Morinda citrifolia* L. (Noni) Seeds against Particulate Matter-Induced Injury

**DOI:** 10.4014/jmb.2407.07062

**Published:** 2024-12-12

**Authors:** Thao Quyen Cao, Hyeongjin Eom, Hyukjin Kim, Ha Yeong Kang, Young Min Park, Sung Keun Jung, Dongyup Hahn

**Affiliations:** 1 Institute of Agricultural Science and Technology, Kyungpook National University, Daegu 41566, Republic of Korea; 2 Department of Integrative Biotechnology, Kyungpook National University, Daegu 41566, Republic of Korea; 3 School of Food Science and Biotechnology, Kyungpook National University, Daegu 41566, Republic of Korea; 4 Research Institute of Tailored Food Technology, Kyungpook National University, Daegu 41566, Republic of Korea

**Keywords:** *Morinda citrifolia*, noni, inflammation, particulate matter, lignans, BEAS-2B cells

## Abstract

*Morinda citrifolia* L. (noni) is native to the tropical and semitropical areas and has been commercially available in health food stores and chain grocery stores specializing in natural foods, recently. Noni seeds are discarded as waste products through the industrial production of noni juice even though their bioactivity components might be a potential source of functional foods. Not many studies of phytochemistry and biological activity have been investigated on noni seeds until now. In this study, the phytochemical investigation of *M. citrifolia* seeds led to the isolation of eight compounds (1-8) including four lignans (5-8). Their chemical structures were elucidated based on extensive spectroscopic analysis as well as the comparison with those reported in the literature. The isolated lignans were then evaluated for their anti-inflammatory activity by the 3-(4,5-dimethylthiazol-2-yl)-2,5-diphynyltetrazolium bromide (MTT) assay in human bronchial epithelium BEAS-2B cells stimulated by 1-nitropyrene. As results, both four isolated lignans displayed high effects on the viability of BEAS-2B cells, indicating promising anti-inflammatory role in the airway disease.

## Introduction

*Morinda citrifolia* L. (noni) is an evergreen shrub belonging to the family Rubiaceae and is widely found in tropical and semitropical areas such as Vietnam, Cambodia, Malaysia, India, and Polynesia. Noni fruit has been in the spotlight as a healthy functional food since the last years of the 20th century [1, 2]. The fruit juice is readily available not only in the countries where it is grown, but also in Europe, the United States, Japan, and other countries as bottled pasteurized juice, either in pure form or mixed with other juices [3]. The reason is that noni fruit presents various bioactive components including lignans, iridoids, coumarins, anthraquinone, flavonoids, polysaccharides, and terpenoids [4, 5]. For instance, nonioside A and tricetin isolated from noni fruit juice exhibited potential inhibition of the lipopolysaccharide (LPS)-induced inflammation in RAW 264.7 macrophages through the nuclear factor kappa-B (NF-κB) and IκB kinase (IKK)α/*β* signalling pathways [6]. Noni’s polysaccharides, which are composed of homogalacturonan and arabinogalactan residues, have been proven to decrease the inflammatory parameters in paw oedema induced *via* histamine, serotonin, dextran, and carrageenan [7]. In addition, phenolic compounds from noni played promising roles in not only anti-oxidative effects but also antiadhesion for AGS cells from *Helicobacter pylori* infection then reduced the risk of gastric diseases [5, 8]. Furthermore, noni seeds were demonstrated as a potential new source of vegetable oil because they contain a huge amount of bioactive unsaturated fatty acids [9, 10]. Air-dried seeds constitute 2.5% of the whole fruit; however, they are discarded as waste products through the industrial production of noni juice. The discard of noni seeds thus adds a lot of pressure on industrial waste treatments. Notwithstanding that, the investigation on phytochemistry and biological activity of noni seeds has been scarce until now [10-13].

Besides, being a major issue for the global community, air pollution has affected the human immune system, causing chronic and acute respiratory disease, lung cancer, chronic bronchitis, and heart diseases. Especially, airborne particulate matter (PM) is one of the most important air pollutants. PM consists of particles characterized in coarse (PM_10_), fine (PM_2.5_), and ultrafine (PM_0.1_) for aerodynamic diameter smaller than 10, 2.5, and 0.1 μm, respectively [14]. On inhalation, PM_10_ is retained in the nasal cavities and upper airways, whereas PM_2.5_ and PM_0.1_ may penetrate deeper into the lung [15]. Fine PM has been widely reported to stimulate an inflammatory response in macrophages through activation of toll-like receptor 4 (TLR4), cyclooxygenase-2 (COX-2), and NF-κB signaling [16]. Additionally, airway chronic inflammation is related to structural changes in the airway wall and parenchyma. During inflammation, airway epithelial cells, as well as macrophages and neutrophils, release interleukin (IL)-6, IL-8, tumor necrosis factor-α (TNF-α), and monocyte chemoattractant protein (MCP)-1, which can cause airway damage [17]. Hence, inhaled PM easily affects the respiratory health, then may cause pulmonary, cardiovascular diseases, inflammation, and lung injury as well as diabetic symptoms [18]. In recent years, natural products have been rich sources of therapeutic agents for chronic inflammation including inflammatory responses related to PM-induced [19, 20]. Phenolic compounds are considered to alleviate PM-induced inflammatory reactions *via* lots of mechanisms, including inflammatory cytokines, reactive oxygen species (ROS), and autophagy [20]. Our previous study has revealed the inhibitory effects of cardamonin, a flavone compound isolated from *Alpinia katsumadai* Heyata, against PM_2.5_-induced lung injury by its ability to regulate TLR2,4-mammalian target of rapamycin (mTOR)-autophagy pathways [21]. The phenanthrenes isolated from *Dioscorea batatas* Decne peel have been revealed to potentially inhibit PM_2.5_-induced endothelial barrier disruption and reduce PM_2.5_-induced lung vascular leakage through the p38 mitogen-activated protein kinase (MAPK) pathway [22].

In this study, our continued efforts to study anti-inflammatory compounds isolated from food products led to the isolation eight of compounds (**1‒8**) from noni seeds. Their chemical structures were elucidated based on extensive spectroscopic analysis as well as the comparison with those reported in the literature. The isolated lignans were then evaluated for their anti-inflammatory activity by the 3-(4,5-dimethylthiazol-2-yl)-2,5-diphynyltetrazolium bromide (MTT) assay in human bronchial epithelium BEAS-2B cells stimulated by 1-nitropyrene (1-NP).

## Materials and Methods

### General Experimental Procedures

Acetonitrile (ACN, reagent grade), water (H_2_O, reagent grade), and methanol (MeOH, reagent grade) were purchased from J.T.Baker (USA). Trifluoroacetic acid (TFA) was purchased from Sigma-Aldrich (USA). Organic solvents such as *n*-butanol, dichloromethane (CH_2_Cl_2_), ethyl acetate (EtOAc), *n*-hexane, and methanol (MeOH) of extra pure grade were purchased from Duksan (Republic of Korea). Nuclear magnetic resonance (NMR) spectra were recorded in methanol-*d*_4_ (δ_H_/δ_C_ = 3.31, 4.87/49.00) using an Ascend 500 spectrometer (Bruker, USA) and the chemical shifts were referenced relative to the residual solvent peaks. Waters 1525 high-performance liquid chromatography (HPLC) (Waters, USA) which was performed on a Hector-A-C18 column (250 × 21.2 mm, 5 μm, RS Tech Co., Republic of Korea) was used for isolation and purification.

### Plant Material

The seeds of *M. citrifolia* were offered from Noni Planet Co. Ltd., (Republic of Korea). The seeds were obtained from plants cultivated in Nakhon Nayok, Thailand.

### Extraction and Isolation

The dried noni seeds (672.83 g) were ground and extracted with 10 L of CH_2_Cl_2_-MeOH (1:1, v/v) at room temperature. The extract was evaporated to dryness, resulting in a crude extract (88.51 g). The crude extract was then suspended in water and partitioned with *n*-hexane, EtOAc, and *n*-butanol, successively, to afford *n*-hexane-(65.85 g), EtOAc- (7.25 g), and *n*-butanol- (6.67 g), and H_2_O- (4.88 g) soluble fractions, respectively. The EtOAc extract was subdivided into seven fractions (E1‒7) using Sephadex LH20 resin (80×400 mm) size exclusion column chromatography (Pharmacia, Sweden) eluting with CH_2_Cl_2_-MeOH (1:1, v/v). Fraction E7 (2.044 g) was purified by HPLC [eluted with an isocratic solvent of 20% ACN and 0.1% TFA in water over 50 min; flow rate: 3 ml/min; UV detection at 280 and 320 nm] to obtain eight compounds **1** (8.6 mg), **2** (4.8 mg), **3** (8.7 mg), **4** (34.4 mg), **5** (1.0 mg), **6** (14.5 mg), **7** (11.8 mg), and **8** (42.3 mg).

*p*-Hydroxybenzoic acid (**1**) ‒ White powder; ^1^H NMR (500 MHz, methanol-*d*_4_) δ (ppm): 7.88 (d, *J* = 8.8 Hz, H-2, H-6), 6.82 (d, *J* = 8.8 Hz, H-3, H-5); ^13^C NMR (125 MHz, methanol-*d*_4_) δ (ppm): 170.2 (C-7), 163.5 (C-4), 133.1 (C-2, C-6), 122.9 (C-1), 116.2 (C-3, C-5). ESI-MS *m/z*: 137.06 [M-H]^-^ (calcd. for C_7_H_5_O_3_: 137.0233).

Vanillic acid (**2**) ‒ Light yellow powder; ^1^H NMR (500 MHz, methanol-*d*_4_) δ (ppm): 7.56 (m, H-2, H-5), 6.84 (d, *J* = 8.7 Hz, H-6), 3.89 (s, OC*H*_3_-3); ^13^C NMR (125 MHz, methanol-*d*_4_) δ (ppm): 170.2 (C-7), 152.8 (C-4), 148.8 (C-3), 125.4 (C-6), 123.2 (C-1), 116.0 (C-2), 114.0 (C-5), 56.5 (OC*H*_3_-3). ESI-MS *m/z*: 167.05 [M-H]^-^ (calcd. for C_8_H_7_O_4_: 167.0339).

*p*-Coumaric acid (**3**) ‒ White solid; ^1^H NMR (500 MHz, methanol-*d*_4_) δ (ppm): 7.60 (d, *J* = 15.9 Hz, H-7), 7.45 (d, *J* = 8.6 Hz, H-3, H-5), 6.80 (d, *J* = 8.6 Hz, H-2, H-6), 6.28 (d, *J* = 15.9 Hz, H-8); ^13^C NMR (125 MHz, methanol-*d*_4_) δ (ppm): 171.1 (C-9), 161.3 (C-4), 146.8 (C-7), 131.2 (C-2, C-6), 127.4 (C-1), 116.9 (C-3, C-5), 115.7 (C-8). ESI-MS *m/z*: 165.14 [M-H]^-^ (calcd. for C_9_H_9_O_3_: 165.0557).

Caffeic acid (**4**) ‒ Yellow solid; ^1^H NMR (500 MHz, methanol-*d*_4_) δ (ppm): 7.53 (d, *J* = 15.9 Hz, H-7), 7.03 (d, *J* = 2.2 Hz, H-2), 6.93 (dd, *J* = 8.1, 2.2 Hz, H-6), 6.78 (d, *J* = 8.1 Hz, H-5), 6.22 (d, *J* = 15.9 Hz, H-8); ^13^C NMR (125 MHz, methanol-*d*_4_) δ (ppm): 171.2 (C-9), 149.6 (C-4), 147.2 (C-3), 146.9 (C-7), 127.9 (C-1), 123.0 (C-6), 116.6 (C-5), 115.7 (C-2), 115.2 (C-8). ESI-MS *m/z*: 179.13 [M-H]^-^ (calcd. for C_9_H_8_O_4_: 179.0339).

Morindolin (**5**) ‒ Pale yellow amorphous powder; ^1^H NMR (500 MHz, methanol-*d*_4_) δ (ppm): 7.57 (d, *J* = 15.9 Hz, H-7'), 7.06 (d, *J* = 1.6 Hz, H-2'), 6.97 (d, *J* = 1.6 Hz, H-6'), 6.82 (d, *J* = 1.9 Hz, H-2), 6.75 (dd, *J* = 8.2, 1.8 Hz, H-5), 6.72 (dd, *J* = 8.2, 1.9 Hz, H-6), 6.27 (d, *J* = 15.9 Hz, H-8'), 5.51 (d, *J* = 6.0 Hz, H-7), 3.81 (m, H-9), 3.49 (q, *J* = 6.0 Hz, H-8); ^13^C NMR (125 MHz, methanol-*d*_4_) δ (ppm): 171.1 (C-9'), 151.2 (C-7'), 147.1 (C-3), 146.6 (C-3'), 146.5 (C-4), 142.8 (C-4'), 134.7 (C-1), 131.1 (C-5'), 129.7 (C-1'), 118.8 (C-6), 118.0 (C-2'), 116.9 (C-6'), 116.4 (C-5), 116.0 (C-8'), 114.0 (C-2), 89.6 (C-7), 65.0 (C-9), 55.2 (C-8). ESI-MS *m/z*: 343.25 [M-H]^-^ (calcd. for C_18_H_15_O_7_: 343.0812).[Fig F1]

(‒)-3,4,3',4'-Tetrahydroxy-9,7'*β*-epoxylignano-7*β*,9'-lactone (**6**) ‒ Brown amorphous powder; ^1^H NMR (500MHz, methanol-*d*_4_) δ (ppm): 6.78 (d, *J* = 2.2 Hz, H-2'), 6.76 (d, *J* = 2.0 Hz, H-2), 6.75 (d, *J* = 8.2 Hz, H-5), 6.71 (d, *J* = 8.1 Hz, H-5'), 6.68 (dd, *J* = 8.1, 2.2 Hz, H-6'), 6.63 (dd, *J* = 8.2, 2.0 Hz, H-6), 5.33 (d, *J* = 3.7 Hz, H-7), 5.04 (d, *J* = 3.8 Hz, H-7'), 4.13 (dd, *J* = 9.3, 7.1 Hz, H-9), 3.92 (dd, *J* = 9.3, 4.4 Hz, H-9), 3.61 (dd, *J* = 9.2, 3.8 Hz, H-8'), 3.23 (m, H-8); ^13^C NMR (125 MHz, methanol-*d*_4_) δ (ppm): 180.0 (C-9'), 147.0 (C-3), 146.8 (C-3'), 146.6 (C-4), 146.3 (C- 4'), 133.3 (C-1'), 132.5 (C-1), 118.8 (C-6), 118.6 (C-6'), 116.8 (C-5), 116.6 (C-5'), 114.3 (C-2'), 114.1 (C-2), 87.3 (C-7), 85.1 (C-7'), 73.8 (C-9), 54.4 (C-8'), 50.9 (C-8). ESI-MS *m/z*: 343.26 [M-H]^-^ (calcd. for C_18_H_15_O_7_: 343.0812).

3,3'-Bisdemethylpinoresinol (**7**) ‒ Pale yellow amorphous powder; ^1^H NMR (500 MHz, methanol-*d*_4_) δ (ppm): 6.80 (d, *J* = 2.0 Hz, H-2, H-2'), 6.74 (d, *J* = 8.2 Hz, H-5, H-5'), 6.68 (dd, *J* = 8.2, 2.0 Hz, H-6, H-6'), 4.62 (d, *J* = 4.3 Hz, H-7, H-7'), 4.19 (dd, *J* = 9.1, 6.8 Hz, H-9, H-9'), 3.78 (dd, *J* = 9.1, 3.5 Hz, H-9, H-9'), 3.06 (m, H-8, H-8'); ^13^C NMR (125 MHz, methanol-*d*_4_) δ (ppm): 146.6 (C-4, C-4'), 146.2 (C-3, C-3'), 134.0 (C-1, C-1'), 119.0 (C-6, C-6'), 116.4 (C-5, C-5'), 114.6 (C-2, C-2'), 87.6 (C-7, C-7'), 72.7 (C-9, C-9'), 55.4 (C-8, C-8'). ESI-MS *m/z*: 329.27 [M-H]^-^ (calcd. for C_18_H_17_O_6_: 329.1019).

Americanoic acid A (**8**) ‒ Pale yellow amorphous powder; ^1^H NMR (500 MHz, methanol-*d*_4_) δ (ppm): 7.56 (d, *J* = 15.9 Hz, H-7'), 7.17 (d, *J* = 2.0 Hz, H-2'), 7.13 (dd, *J* = 8.4, 2.0 Hz, H-6'), 6.99 (d, *J* = 8.4 Hz, H-5'), 6.87 (d, *J* = 1.9 Hz, H-2), 6.81 (d, *J* = 8.1 Hz, H-5), 6.78 (dd, *J* = 8.1, 1.9 Hz, H-6), 6.32 (d, *J* = 15.9 Hz, H-8'), 4.84 (d, *J* = 8.1 Hz, H-7), 4.05 (ddd, *J* = 7.7, 4.5, 2.5 Hz, H-8), 3.70 (dd, *J* = 12.4, 2.5 Hz, H-9), 3.49 (dd, *J* = 12.4, 4.5 Hz, H-9); ^13^C NMR (125 MHz, methanol-*d*_4_) δ (ppm): 171.1 (C-9'), 147.4 (C-4), 147.3 (C-3), 146.8 (C-4'), 146.0 (C-7'), 145.7 (C-3'), 129.5 (C-1), 129.4 (C-1'), 123.3 (C-6'), 120.6 (C-6), 118.6 (C-5'), 117.8 (C-2'), 117.6 (C-8'), 116.5 (C-5), 115.7 (C- 2), 80.5 (C-8), 77.7 (C-7), 62.1 (C-9). ESI-MS *m/z*: 343.26 [M-H]^-^ (calcd. for C_18_H_15_O_7_: 343.0812).

### MTT Assay

BEAS-2B cells, human bronchial epithelium, were seeded (3 × 10^4^ cells/ml) on a 96-well plate and allowed to reach 70-80% confluency [23]. The medium was changed with *M. citrifolia* seeds lignans contained medium. After 1 h, the cells were treated with 50 μM 1-NP (TCI Chemicals, Japan) and then incubated for 24 h. Afterward, 10 μl of the 3-(4,5-dimethyl-2-thiazolyl)-2,5-diphenyl-2H-tetrazolium bromide (MTT) solution (5 mg/ml)(Sigma-Aldrich, USA) was added to each well and incubated for 4 h. The supernatant of the wells was discarded, then the crystals were dissolved with 100 μl of dimethyl sulfoxide (DMSO) (Sigma-Aldrich). Following shaking for 30 min, the absorbance of the plate was measured at 595 nm by a microplate reader (Bio-Rad Inc., USA).

## Results and Discussion

### Chemical Structure Identification of the Isolated Compounds

**Fig. 2:**
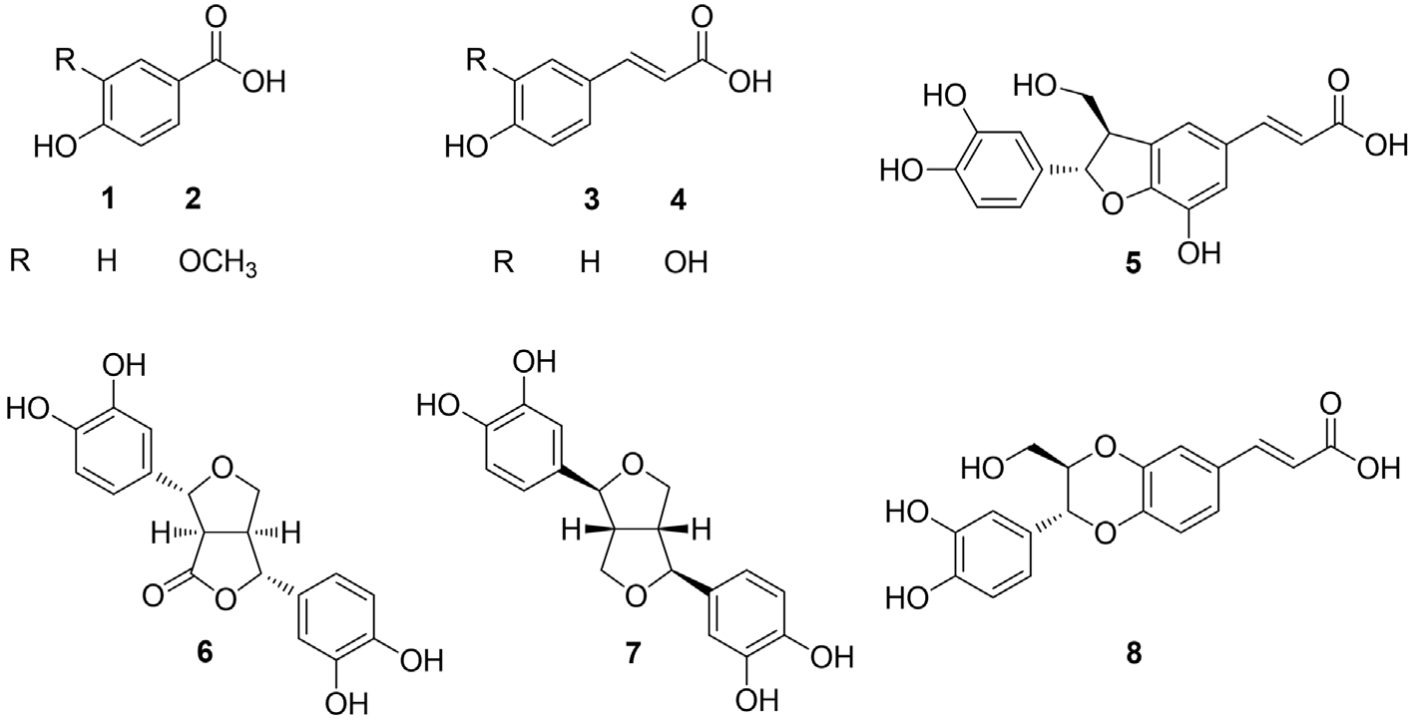
Chemical structures of compounds (1‒8) isolated from *M. citrifolia* seeds.

From the ethyl acetate soluble fraction, eight phenolics (**1**‒**8**) were isolated by efficient chromatographic separation techniques. Their chemical structures were elucidated as *p*-hydroxybenzoic acid (**1**) [24], vanillic acid (**2**) [25], *p*-coumaric acid (**3**) [24, 25], caffeic acid (**4**) [25], morindolin (**5**) [26], (‒)-3,4,3',4'-tetrahydroxy-9,7'*β*-epoxylignano-7*β*,9'-lactone (**6**) [27], 3,3'-bisdemethylpinoresinol (**7**) [26], and americanoic acid A (**8**) [26] based on the comparison of their spectral data with values reported in the literature (Fig. 2).

Compound **1** was obtained as a white powder and exhibited a molecular ion peak at [M-H]^-^ *m/z* 137.06 in LRESI-MS. Compound **1** was identified based on aromatic proton signals at δ_H_ 7.88 (d, *J* = 8.8 Hz, H-2, H-6) and 6.82 (d, *J* = 8.8 Hz, H-3, H-5). The ^13^C NMR spectrum revealed a carboxylic carbon at δ_C_ 170.2 and the signals of six sp2 carbons (δ_C_ 163.5, 133.1, 122.9, and 116.2). The structure was characterized as *p*-hydroxybenzoic acid by comparison of MS value and NMR data with a previous study (24).

Compound **2** was isolated as a light-yellow powder and revealed a molecular ion peak at [M-H]^-^ *m/z* 167.05 in LRESI-MS. The ^1^H NMR spectrum of **2** displayed three aromatic protons at δ_H_ 7.56 (m, H-2, H-5) and 6.84 (d, *J* = 8.7 Hz, H-6), and a methoxy group at δ_H_ 3.89 (s, OC*H*_3_-3). The ^13^C NMR spectrum showed eight signals corresponding to a carboxylic carbon (δ_C_ 170.2), six sp2 carbons (δ_C_ 152.8, 148.8, 125.4, 123.2, 116.0, and 114.0), and a methoxy carbon (δ_C_ 56.5), indicated **2** to be vanillic acid *via* the comparison of MS value and NMR data with a previous report [25].

Compound **3** was obtained as a white solid and exhibited a molecular ion peak at [M-H]^-^ *m/z* 165.14 in LRESI-MS. Compound **3** was characterized by *trans* form protons of a double bond (δ_H_ 7.60 and 6.28, 1H, d, *J* = 15.9 Hz, respectively) and the signals of *para*-substituted aromatic benzene ring (δ_H_ 7.45 and 6.80). The ^13^C NMR spectrum of **3** showed nine signals including a carboxylic carbon (δ_C_ 171.1), six sp2 carbons (δ_C_ 161.3, 146.8, 131.2, 127.4, 116.9, and 115.7). Thus, the structure of **3** was established as *p*-coumaric acid by comparison of the obtained MS value and NMR data with previous reports [24, 25].

Compound **4** was obtained as a yellow solid and displayed a molecular ion peak at [M-H]^-^ *m/z* 179.13 in LRESI-MS. The 1H and ^13^C NMR spectra of **4** indicated that its structure differed from that of **3** by one more hydroxyl group. In the ^1^H NMR spectrum of **4**, two *trans* form protons of a double bond (δ_H_ 7.53 and 6.22, 1H, d, *J* = 15.9 Hz, respectively) and three signals of one ABX-type benzene ring [δ_H_ 7.03 (d, *J* = 2.2 Hz, H-2), 6.93 (dd, *J* = 8.1, 2.2 Hz, H-6), and 6.78 (d, *J* = 8.1 Hz, H-5)] were observed. The ^13^C NMR spectrum of **4** displayed a carboxylic carbon (δ_C_ 171.2), six sp^2^ carbons (δ_C_ 149.6, 147.2, 146.9, 127.9, 123.0, 116.6, 115.7, and 115.2). Thus, the structure of **4** was established as caffeic acid by comparison of the obtained MS value and NMR data with a previous study [25].

Compound **5** was obtained as a pale-yellow amorphous powder and exhibited a molecular ion peak at [M-H]^-^*m/z* 343.25 in LRESI-MS. The ^1^H NMR spectrum of **5** revealed the signals of *trans* double bond moiety (δ_H_ 7.57 and 6.27, 1H, d, *J* = 15.9 Hz, respectively), *meta*-coupled proton signals (δ_H_ 7.06 and 6.97, 1H, d, *J* = 1.6 Hz, respectively), an ABX coupling system [δ_H_ 6.82 (d, *J* = 1.9 Hz, H-2), 6.75 (dd, *J* = 8.2, 1.8 Hz, H-5), and 6.72 (dd, *J* = 8.2, 1.9 Hz, H-6)], an oxygen-bearing methine signal at δ_H_ 5.51, and a methylene group at δ_H_ 3.81, which were coupled to a methine proton at δ_H_ 3.49. The ^13^C NMR spectrum of **5** showed a carboxylic, seven quaternary, one methylene, and nine methine carbons, of which six oxygenated carbons were observed at δ_C_ 147.1, 146.6, 146.5, 142.8, 89.6, and 65.0. By comparing the NMR data and MS value of **5** with those of the previous report, the structure of **5** was elucidated as morindolin [26].

Compound **6** was obtained as a brown amorphous powder and revealed a molecular ion peak at [M-H]^-^ *m/z* 343.26 in LRESI-MS. The ^13^C NMR spectrum of **6** displayed the signals of a carboxylic, a methylene, six aromatic methine, four methine, and six quaternary carbons, including six oxygenated carbons at δ_C_ 147.0, 146.8, 146.6, 146.3, 87.3, and 85.1. The ^1^H NMR spectrum indicated the existence of two 1,3,4-trisubstituted benzene systems (δ_H_ 6.78, 6.76, 6.75, 6.71, 6.68, and 6.63), two methine protons (δ_H_ 3.61 and 3.23), two oxymethine signals (δ_H_ 5.33 and 5.04), and a pair of oxymethylene protons (δ_H_ 4.13 and 3.92). The structure of **6** was established as (‒)-3,4,3',4'-tetrahydroxy-9,7'*β*-epoxylignano-7*β*,9'-lactone by comparing the MS value and NMR data with a previous report [27].

Compound **7** was collected as a pale-yellow amorphous powder and displayed a molecular ion peak at [M-H]^-^*m/z* 329.27 in LRESI-MS. In the ^1^H NMR spectrum, the presence of 1,3,4-trisubstituted benzene systems (δ_H_ 6.80, 6.74, and 6.68), two methine protons (δ_H_ 3.06), two oxymethine signals (δ_H_ 4.62), and two pairs of oxymethylene protons (δ_H_ 4.19 and 3.78) were deduced. The ^13^C NMR spectrum of **7** revealed six signals corresponding to three quaternary (δ_C_ 146.6, 146.2, and 134.0), a methylene (δ_C_ 72.7), and five methine carbons (δ_C_ 119.0, 116.4, 114.6, 87.6, and 55.4) representing half of the total carbon atoms estimated from MS value. Consequently, compound **7** was suggested consisting of two identical units. The structure of **7** was elucidated as 3,3'-bisdemethylpinoresinol by the comparison of MS value and NMR data with a previous study [26].

Compound **8** was isolated as a pale-yellow amorphous powder and exhibited a molecular ion peak at [M-H]^-^ *m/z* 343.26 in LRESI-MS. The ^1^H NMR of **8** revealed the presence of two 1,3,4- trisubstituted benzene rings (δ_H_ 7.17, 7.13, 6.99, 6.87, 6.81, and 6.78), a *trans* double bond (δ_H_ 7.56 and 6.32), one 1,4-dioxane ring (δ_H_ 4.84 and 4.05), and an oxymethylene at δ_H_ 3.70 and 3.49. The ^13^C NMR spectrum deduced the moiety of a carboxylic, six quaternary, one oxymethylene, and ten methine carbons, of which seven oxygenated carbons were observed at δ_C_ 147.4, 147.3, 146.8, 145.7, 80.5, 77.7, and 62.1. By comparing the NMR data and MS value of **8** with those of the previous study, the structure of **8** was elucidated as americanoic acid A [26].

### Effects of Isolated Lignans on PM_2.5_-Induced Lung Damage

Polycyclic aromatic hydrocarbons (PAHs), associated with the toxic substances absorbed on PM, mainly originate from incomplete combustion processes including biomass burning, coal combustion, and vehicle exhaust, and are ubiquitous environmental contaminants. PAHs could increase health risks to be higher than currently accepted due to the production of more toxic derivatives from a series of chemical reactions in the atmosphere [28]. 1-NP is an important subgroup of PAHs present in urban air pollutants and was reported to cause damage to BEAS-2B cells [29]. In addition, lignans are widely present in a range of plant kingdoms including edible plants and represent an enormous class of bioactive compounds [30, 31]. They are one of the common secondary metabolite classes in noni, distinguished depending on the pattern of additional bridging between two phenylpropanoid C6-C3 units at carbons *β* and *β*' [27, 32]. Previous publications have proven the promising effects of lignans in the inhibition of airway inflammatory diseases. As evidence, sauchinone, a lignan presented in *Saururus chinensis*, significantly diminished the production of inflammatory mediators in lungs subjected to LPS *via* MAPK pathways [33]. Lignans from *Magnolia fargesii* have been revealed the potential for the inhibition of airway inflammation diseases through the suppression of epidermal growth factor receptor (EGFR) [34]. Another example of sesamin has been proven by alleviating PM_2.5_-induced lung injury, alveolar permeability, and oxidative damage, and protecting against PM_2.5_-induced lung apoptosis in rats [35].

**Fig. 3:**
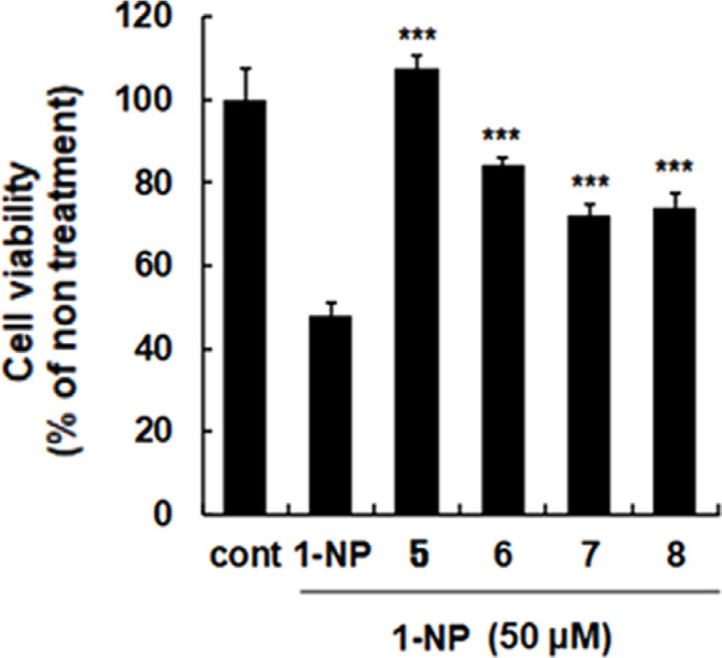
The effects of isolated lignans (5‒8) on cell viability. BEAS-2B cells were incubated to reach 70-80% confluency. Cell viability was determined using a MTT assay. The cells were pretreated for 1 h with isolated lignans and stimulated for 24 h with 1-NP. Bars represent means ± standard deviation of three independent experiments. ***p < 0.001 compared with 1-NP-treated group.

In the present study, to identify the bioactive components from noni seeds, the cytotoxicity of isolated lignans (**5‒8**) on BEAS-2B cells stimulated by 1-NP was evaluated. As results in Fig. 3, all the isolated lignans displayed cell viability percentages of over 60%. Among them, compound **5** showed the most effect with over 100% cell viability. Previous studies revealed that *M. citrifolia*’s markers, **5** and **8**, showed the potential effects on the inhibition of copper-induced low-density lipoprotein oxidation [26] and fatty acid amide hydrolase and monoacylglycerol lipase in vitro [36]. Morindolin (**5**), one of the specific markers of noni, exhibited noticeable activity on the viability of BEAS-2B cells stimulated by 1-NP in our study; nevertheless, there is still limited investigation on its bioactivity up to now. Americanoic acid A (**8**) has displayed weak inhibitory effects on the protein tyrosine phosphatase 1B (PTP1B) enzyme with IC_50_ value over 30 μM [27]. Liu *et al*. reported that compound **6**, a furofuran lignan, displayed the remarkable inhibition of nitric oxide (NO) production induced by LPS in RAW 264.7 cells with an IC_50_ value of 4.6 μM [4] Another isolated furofuran lignan, compound **7**, was reported to be significantly potent in protecting rat hepatocytes and HepG2 cells against ethanol-induced oxidative stress [37], inhibiting melanogenesis *via* the suppression of tyrosinase expression resulting from the down regulation of p38 MAPK phosphorylation [38], and inhibiting NO production in LPS-activated BV2 microglia cells [39]. 3,3'-Bisdemethylpinoresinol (**7**) also prominently affected copper-induced low-density lipoprotein oxidative inhibition with an IC_50_ value of 1.057 μM [26].

To the best of our knowledge, the present work is the first investigation on the potential effects of noni lignans on PM_2.5_-induced lung injury until now. These results proved the potential effects on the anti-inflammatory activity of lignans isolated from noni seeds, thereby suggesting the promising role of noni seeds in the development of biomaterials and lignans are their active composites. Also, these findings provide the direction of research and application of the discarded noni seeds therefore alleviating pressure on industrial waste treatments in general and on the environment in particular.

## Figures and Tables

**Fig. 1 F1:**
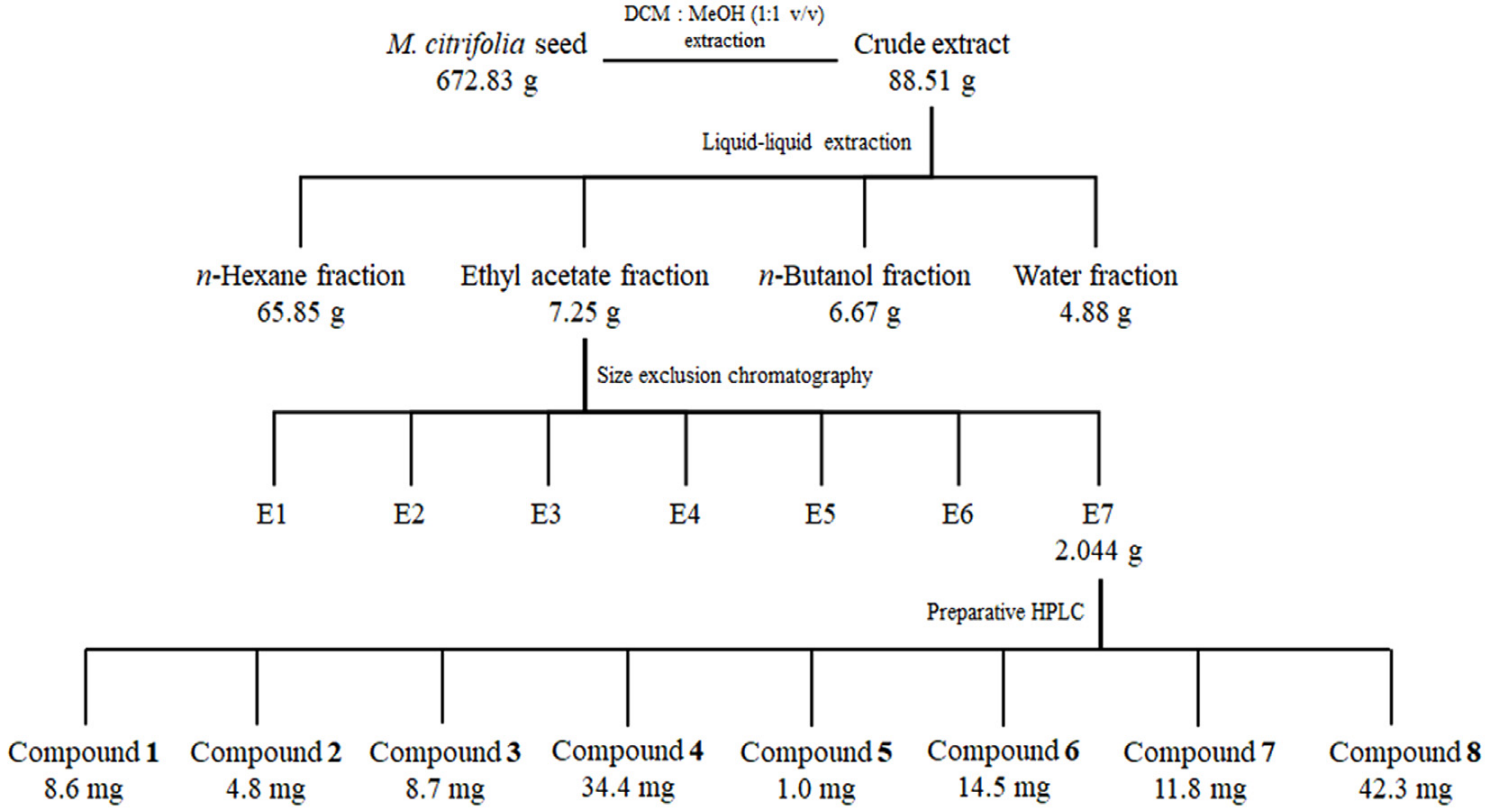
Isolation scheme of *M. citrifolia* seeds.
